# Editorial: Microbial co-cultures: a new era of synthetic biology and metabolic engineering

**DOI:** 10.3389/fmicb.2023.1235565

**Published:** 2023-06-23

**Authors:** Durgesh Kumar Jaiswal, Jay Prakash Verma, Tarun Belwal, Arthur Prudêncio De Araujo Pereira, Avinash Bapurao Ade

**Affiliations:** ^1^Department of Botany, Savitribai Phule Pune University, Pune, Maharashtra, India; ^2^Plant-Microbe Interaction Lab, Institute of Environment and Sustainable Development, Banaras Hindu University, Varanasi, Uttar Pradesh, India; ^3^Texas A&M University, College Station, TX, United States; ^4^Federal University of Ceará, Soil Science Department, Soil Microbiology Laboratory, Fortaleza, Ceará, Brazil

**Keywords:** synthetic microbial consortia, metabolic engineering, sustainable agriculture, industrial, environment

This special issue explores the field of microbial engineering consortia and its potential applications in agriculture, industrial, and environmental restoration. Microbial consortia are communities of diverse groups of microorganisms that collaborate to achieve collective goals. By harnessing symbiotic interactions and synergistic behaviors, scientists aim to unlock various applications beyond what single organisms can accomplish. In the realm of agriculture, microbial consortia hold tremendous promise. They can enhance nutrient availability, suppress diseases, improve soil health, promote plant growth, manage abiotic stress, increase crop productivity, and support sustainable farming methods. However, further research is needed to optimize consortia composition, stability, and functionality for multiple crops' productivity, environmental sustainability, and cost-effectiveness concerns. Microbial consortia also show immense potential for ecological restoration and revitalization. They can be utilized for bioremediation of contaminated sites, restoration of soil health, wastewater treatment, ecological restoration, climate change mitigation, and environmental health assessment. However, similar to agricultural applications, further research is necessary to optimize their application in specific contexts. This special issue features cutting-edge research articles and reviews that delve into various aspects of microbial engineering consortia. The articles cover topics such as industrial enzyme production, plant growth promotion under stress conditions, utilizing CO_2_ as a feedstock for chemical manufacturing, understanding community dynamics and phenotypes, function-driven co-cultures for tobacco quality improvement, desiccation-tolerant bacteria for crop resilience, artificial plant-bacteria systems, biocontrol of crop diseases, and the development of nano-pesticides. Additionally, the issue explores using bacterial biosensors for environmental pollution detection and creating synthetic consortia for the sustainable production of ethylene and isoprene. Overall, this special issue provides insights into the exciting and rapidly advancing field of microbial engineering consortia and highlights the potential applications, challenges, and prospects in agricultural fields.

Synthetic biology has emerged as a transformative field, revolutionizing the way we approach complex biological systems. Synthetic biology aims to design, construct, and manipulate biological components to create novel functionalities and address real-world challenges (Andrianantoandro et al., 2006; Liang et al., 2022). Over the years, researchers made remarkable progress in engineering individual microorganisms to perform specific tasks. However, the true potential of synthetic biology lies in the collaborative efforts of microbial consortia–a new frontier that holds immense promise in various domains (Brenner et al., 2008; Ben Said and Or, 2017).

In this special issue, we delve into the exciting world of engineering microbial consortia and explore its potential applications, challenges, and prospects in agriculture and the environment. Microbial consortia refer to communities of diverse microorganisms that interact and work together to achieve collective objectives. By harnessing symbiotic interactions and synergistic behaviors, scientists' main aim is to unlock a range of applications that go beyond what single organisms can achieve (Hays et al., 2015; Liu et al., 2023).

Indeed, microbial consortia hold tremendous promise in agriculture. The use of these communities of microorganisms can revolutionize agricultural practices by improving nutrient availability (Yadav et al., 2014; Shukla et al., 2020; Vishwakarma et al., 2020; Jaiswal et al., 2022b; Islam et al., 2023); disease suppression (Sarma et al., 2015; Gómez Expósito et al., 2017; Dheeman et al., 2023); soil health (Khan et al., 2023; Nabi, 2023); plant growth promotion (Verma et al., 2014a, 2023; Méndez-Bravo et al., 2023); crop protection (Jaiswal et al., 2022a); management abiotic stress (Krishna et al., 2022; Prajapati et al., 2022; Hett et al., 2023; Mahreen et al., 2023); sustainable crop productivity (Singh et al., 2016; Mukherjee et al., 2022; Kaushal et al., 2023; Paravar et al., 2023), and promoting sustainable farming methods (Jha et al., 2013; Kong et al., 2018; Gehlot et al., 2021). It is worth noting that engineering microbial consortia for agricultural applications is still in its early stages. Further research is needed to optimize consortia composition, stability, and functionality for multiple crops under different environments and agroclimatic regions. Additionally, the scalability and cost-effectiveness of implementing microbial consortia in agricultural systems need consideration.

In addition, one more area where microbial consortia have immense potential for environmental restoration and revitalization (Verma et al., 2014b; Abhilash et al., 2016; Pankaj and Pandey, 2022; Singh et al., 2023). The complex interactions within these communities can be harnessed to address ecological challenges and restore ecosystems (Liang et al., 2022). Researchers explore the study in which microbial consortia show promise in environmental revival: bioremediation of contaminated sites (Jaiswal et al., 2019; Zhang et al., 2020; Li et al., 2023; Wu et al., 2023); restoration of Soil Health (Lebrun et al., 2021); wastewater Treatment (Mazzucotelli et al., 2014; Heredia et al., 2022); ecological restoration (Singh et al., 2023); climate change mitigation (Hamilton et al., 2016; Silverstein et al., 2023) and monitoring and environmental health assessment (Bhatia et al., 2018; Chandran et al., 2020). The field of engineering microbial consortia for environmental revival is still evolving, and further research is needed to optimize their application in specific contexts.

However, in this special issue, we have a collection of 13 cutting-edge research articles and two review articles that shed light on the diverse aspects of engineering microbial consortia and their functional attributes for agricultural productivity and environmental & industrial sustainability.

## Engineering microbial consortia: sustainable agriculture

The engineering of microbial consortiums for sustainable agriculture has great promise for increasing yields while reducing negative environmental effects. These coalitions can enhance nutrient cycling, disease control, soil health, and plant resilience by harnessing the strength of microbial interactions. However, the full potential of microbial consortia in attaining sustainable and resilient agricultural systems relies on further study and innovation in this sector. Furthermore, the widespread use of engineered microbial consortia in sustainable agriculture still faces obstacles like scaling up from laboratory to field conditions, ensuring stability and persistence of the consortia, and addressing regulatory and ethical considerations. In this Research Topic, the following articles collection addresses the current need for research to promote sustainable agriculture.

Gupta et al. claimed that integrated and balanced nutrient management inspired the trial. Organic manures with N_2_-fixing, P-, and K-solubilizing microbial inoculants improved the crop growth, root development, and soil health. The study's findings may help to manage crop wastes and animal bio-products by encouraging microbial consortia and organic manure-based agro-industries, vital to microbial inoculants and integrated nutrient management (INM). Thus, the study would sustain agricultural growth by producing high-quality crops, sustaining agro-biodiversity, and making soils healthy, fertile, and productive.

Zeyad et al. observed that applying *Streptomyces araujoniae* strains (TN11 and TN19) individually and as a consortium reduces wilt severity in chickpea plants. Reduction in disease development was supported by the production of antifungal metabolites at a higher level by *S araujoniae* strains. The results on disease development, plant growth parameters, physiological and biochemical parameters, and gene expression studies suggest that the consortium of TN11 and TN19 can act as an efficient biocontrol tool against chickpea wilt caused by *Fusarium oxysporum* f. sp. ciceris (Foc).

Mageshwaran et al. recorded that the bacterial endophytes had biocontrol capability. Three promising isolates inhibited all three soil-borne fungal infections (*R. solani*, S. *rolfsii*, and *F. oxysporum* f.sp. ciceri) *in-vitro*. *Bacillus subtilis* strains TRO4 and CLO5-treated chickpea seeds increased plant development and reduced complex wilt disease incidence in the in-planta assay. This study explores chickpea plant complex wilt disease management with possible endophytes. Eco-friendly wilt disease management would improve plant and soil health.

Shahid et al. studied that natural and human-induced stresses affect crop plant growth and yield. Plant stress hormone ethylene can hinder growth and survival. However, ACC deaminase, an enzyme that decreases ethylene levels, can reduce stress and increase crop yield. According to the researchers, plant growth-promoting rhizobacteria (PGPR) with ACC deaminase activity enhance plant growth under unfavorable conditions like salt stress, water deprivation, extreme temperatures, waterlogging, heavy metals, pesticides, and organic pollutants. They also used molecular biotechnology and omics methods like proteomics, transcriptomics, metagenomics, and NGS to find and characterize stress-tolerant PGPR strains that produce ACC deaminase. These microorganisms can help crops flourish and tolerate harsh environmental conditions.

Shankar and Prasad observed that desiccation-tolerant plant growth-promoting rhizobacteria (DT-PGPR) may reduce the deleterious effects of water stress on wheat yield and physiology. Under desiccation stress, five DT-PGPR isolates—*Enterobacter cloacae* BHUAS1, *Bacillus cereus* BHUAS2, *Bacillus megaterium* BHUIESDAS3, 4, and 5—grew and promoted plant growth. In a pot experiment with water-stressed wheat plants, inoculation with Enterobacter cloacae BHUAS1, *Bacillus cereus* BHUAS2, and *Bacillus megaterium* BHUIESDAS3 improved growth, chlorophyll and carotenoid content, antioxidant enzyme activity, and oxidative stress markers. These DT-PGPR strains may boost wheat growth and yield under water stress.

## Engineering microbial consortia: industrial application

The versatility and potential of engineered microbial consortia in various industrial applications. Researchers and engineers can develop innovative solutions for sustainable production (biofuel production, biodegradable plastic production, antibiotics, enzymes, and bioactive compounds), waste management, and resource utilization by leveraging the interactions and synergies among microorganisms. Continued research and technological advancements in this field promise to unlock further opportunities for industrial applications of microbial consortia. Under this Research Topic, the following collected articles have to address research industrial applications by harnessing the multi-facility potential of microbial consortia.

Benito-Vaquerizo et al. explored using one-carbon (C1) compounds, particularly CO_2_, as a sustainable feedstock for producing valuable chemicals. They demonstrated that acetogens, microorganisms capable of utilizing CO_2_/H_2_ gas mixtures, can convert these substrates into ethanol and acetate. A wider range of products, including butyrate, can be obtained by co-cultivating acetogens with solventogens, which produce medium-chain fatty acids (MCFA) and alcohols. Their study employed metabolic modeling to design a co-culture of specific microbial species, and experimental validation confirmed the feasibility and potential of utilizing such microbial consortia to produce chemicals from renewable resources.

Lobo-Moreira et al. stated that the industrial microalgae-fungi consortia use hinders research. Science explored fungi's involvement in microalgae study. Database searches found 1,452 publications from 1950 to 2020, rising sharply after 2006. Chinese writers and organizations topped publications rankings in China, the US, and Germany. Microalgae-fungi consortia focused on biodiesel, lipid accumulation, anaerobic digestion, and biogas upgrading. Industrial-scale microalgae-based biofuel biorefineries still need work. Microalgae-fungi applications for greenhouse gas abatement are intriguing ideas.

Pawar et al. provides a comprehensive overview of the expanding use of microbial enzymes to replace chemical processes in industrial applications. Due to their versatility and alkaline stability, proteases, especially fungi-derived ones, are used in many sectors. Fungi have a wider range of proteases and are safer for industrial application than bacterial alkaline proteases. This study discusses the classification, generation, and usage of alkaline proteases from diverse fungi, emphasizing more research on alkaline-tolerant and alkaliphilic fungi and their biotechnological potential.

Wu et al. conducted a study to address the need for improved quality in flue-cured tobacco (FCT) by developing a function-driven co-culture of microorganisms. They identified *Bacillus kochii* SC, which reduces tobacco irritation, and *Filobasidium magnum* F7, which enhances aroma and flavor. Co-cultivating these strains at a specific ratio significantly improved FCT quality within just 2 days, surpassing the efficiency and cost of the traditional spontaneous aging process that takes over 2 years. The study demonstrated the effectiveness of the function-driven co-culture in achieving desired tobacco quality and suggested its potential application in the tobacco industry through bioaugmentation.

## Engineering microbial consortia: environmental application

It provides innovative solutions to a variety of environmental problems. These consortia can help rehabilitate damaged areas, sustainable waste management, and ecological restoration by using the collective capacities of various microorganisms.

Barman et al. explained that *Bemisia tabaci* (whitefly) is a major agricultural pest and plant virus vector. The genetic variation of whiteflies from West Bengal, India, revealed cryptic species. Most endosymbionts were *Arsenophonus*. Thiamethoxam was the most susceptible pesticide. Insecticide resistance genes were upregulated, and *Arsenophonus* and Wolbachia titers were linked with resistance. The study implies that symbiont-oriented management could reduce whitefly populations and pesticide resistance.

Cui et al. found that ethylene and isoprene production, necessary for polymers and materials, is inefficient and polluting; thus, generating these compounds from biomass or CO_2_ is essential for a more sustainable economy. This study created artificial consortia of *E. coli* strains producing ethylene, isoprene, and sucrose-producing cyanobacteria. In addition, they increased sucrose yields by introducing the sucrose transport gene into cyanobacteria strains, which shuttled carbon and electrons amongst community components. These artificial consortia produced more ethylene and isoprene than *E. coli* cultures alone, indicating their benefit to platform chemical synthesis.

Danish et al. synthesized silver nanoparticles (Ag-NPs) from *Cassia fistula* leaf extract and test them as nano-pesticides against main tomato phytopathogens. The synthesized Ag-NPs were spherical using diverse methods with an average diameter of 16 nm. Ag-NPs reduced bacterial and fungal pathogen viability, morphology, and biofilm formation. They also increased phytopathogen-challenged tomato plant growth, physiological indices, and antioxidant enzyme activity. These findings suggest that *Cassia fistula* leaf extract-synthesized Ag-NPs could be useful and sustainable nano-pesticides in green agriculture for disease management and plant health.

Guo et al. observed that bioavailable lead is essential for forecasting ecological risks and protecting human health from environmental lead pollution. This work develops low-equipment environmental pollution biosensors using bacterial biosensors with visual pigment output signals. Reconstructing *Escherichia coli's* PbrR-based Pb(II) sensing element's anthocyanin biosynthesis pathway creates a metabolic-engineered biosensor. This biosensor uses colored anthocyanin derivatives as a visual signal and better detect low Pb(II) concentrations than fluorescent protein-based biosensors. The biosensor also has a large linear dose-response range and is unaffected by organic and inorganic water samples. These results show that the metabolic engineering of natural colorants can create visible, sensitive, and cost-effective bacterial biosensors for heavy metal pollution detection.

Astafyeva et al. created an artificial plant-bacteria system utilizing the microalga *Micrasterias radians* MZCH 672 and *Dyadobacter* sp. HH091. In pure algal cultures, Bacteroidota phylum member HH091 greatly increased microalga growth. They found HH091 genes linked to the type IX secretion system (T9SS) in transcriptome and genome analysis, which may be involved in the bacterium-microalga interaction. This research will aid symbiotic microalgal–bacteria studies. Additionally, the co-farming of microalgae and bacteria will have favorable commercial and environmental effects on microalgal cultivation.

McClure et al. conducted a comprehensive study on a chitin-degrading synthetic community of soil microbes and examined how it responds to losing key members. They found that the absence of a primary degrader, even if other members can potentially fill the same metabolic niche, led to a collapse in community growth and respiration. The study highlighted that diversity and redundancy alone are insufficient for community resilience; the emerging species must possess the same sharing phenotype and interaction network to support community growth. The findings contribute to our understanding of how keystone species and genomic redundancy changes can impact soil microbiomes and their ability to adapt to a changing climate.

As we embark on this new frontier in synthetic biology, interdisciplinary collaborations, and knowledge sharing will be vital. Engineers, biologists, computer scientists, and ethicists must come together to address the scientific, technical, and ethical challenges that lie ahead. Additionally, interdisciplinary collaborations between biologists, ecologists, and environmental scientists are essential for unlocking the full potential of microbial consortia in environmental restoration. By embracing this innovative approach, we can move toward a more sustainable and environmentally conscious agricultural sector that meets the growing demands for food production while preserving our natural resources. We are excited to present this special issue on Engineering Microbial Consortia, and we hope that the articles published in this issue will inspire researchers, policymakers, and industrial partners to further explore and unlock the potential of complex biological communities for multiple applications. Together, we can harness the power of microbial consortia to address global challenges, improve human health, and pave the way for a more sustainable future.

## Author contributions

DJ, JV, TB, AP, and AA contributed to writing this Editorial on the Research Topic and approved it for publication. All authors contributed to the article and approved the submitted version.

## References

[B1] Abhilash P. C. Dubey R. K. Tripathi V. Gupta V. K. Singh H. B. (2016). Plant growth-promoting microorganisms for environmental sustainability. Tren. Biotechnol. 34, 847–850. 10.1016/j.tibtech.2016.05.005 27265889

[B2] Andrianantoandro E. Basu S. Karig D. K. Weiss R. (2006). Synthetic biology: new engineering rules for an emerging discipline. Mol. Syst. Biol. 2, 2006–0028. 10.1038/msb4100073 16738572 PMC1681505

[B3] Ben Said S. Or D. (2017). Synthetic microbial ecology: engineering habitats for modular consortia. Front. Microbiol. 8, 1125. 10.3389/fmicb.2017.01125 28670307 PMC5472676

[B4] Bhatia S. K. Bhatia R. K. Choi Y. K. Kan E. Kim Y. G. Yang Y. H.. (2018). Biotechnological potential of microbial consortia and future perspectives. Crit. Rev. Biotechnol. 38, 1209–1229. 10.1080/07388551.2018.1471445 29764204

[B5] Brenner K. You L. Arnold F. H. (2008). Engineering microbial consortia: a new frontier in synthetic biology. Trends Biotechnol. 26, 483–489. 10.1016/j.tibtech.2008.05.004 18675483

[B6] Chandran H. Meena M. Sharma K. (2020). Microbial biodiversity and bioremediation assessment through omics approaches. Front. Environ. Chem. 1, 570326. 10.3389/fenvc.2020.570326

[B7] Dheeman S. Kumar M. Maheshwari D. K. (2023). “Beneficial microbial mixtures for efficient biocontrol of plant diseases: impediments and success,” in Sustainable Agrobiology: Design and Development of Microbial Consortia. (Singapore: Springer Nature Singapore), pp. 23-40. 10.1007/978-981-19-9570-5_2

[B8] Gehlot P. Pareek N. Vivekanand V. (2021). Development of biofertilizers and microbial consortium an approach to sustainable agriculture practices. Plant Soil Microbe. Trop. Ecosyst. 3, 315–348. 10.1007/978-981-16-3364-5_15

[B9] Gómez Expósito R. De Bruijn„ Postma I. J. Raaijmakers J. M. (2017). Current insights into the role of rhizosphere bacteria in disease suppressive soils. Front. Microbiol. 8, 2529. 10.3389/fmicb.2017.02529 29326674 PMC5741648

[B10] Hamilton C. E. Bever J. D. Labbe J. Yang X. Yin H. (2016). Mitigating climate change through managing constructed-microbial communities in agriculture. Agric. Ecosyst. Environ. 216, 304–308. 10.1016/j.agee.2015.10.006

[B11] Hays S. G. Patrick W. G. Ziesack M. Oxman N. Silver P. A. (2015). Better together: engineering and application of microbial symbioses. Curr. Opin. Biotechnol. 36, 40–49. 10.1016/j.copbio.2015.08.008 26319893

[B12] Heredia M. Layedra-Almeida A. P. Torres Y. Toulkeridis T. (2022). Evaluation of a microbial consortium and selection of a support in an anaerobic reactor directed to the bio-treatment of wastewater of the textile industry. Sustainability 14, 8889. 10.3390/su14148889

[B13] Hett J. Döring T. F. Bevivino A. Neuhoff D. (2023). Impact of microbial consortia on organic maize in a temperate climate varies with environment but not with fertilization. Eur. J. Agron. 144, 126743. 10.1016/j.eja.2023.126743

[B14] Islam M. M. Rengel Z. Storer P. Siddique K. H. Solaiman Z. M. (2023). Microbial consortium inoculant and rock mineral fertiliser differentially improved yield and nutrient uptake of industrial hemp (*Cannabis sativa* L.) varieties. Ind. Crop. Prod. 197, 116599. 10.1016/j.indcrop.2023.116599

[B15] Jaiswal D. K. Gawande S. J. Soumia P. S. Krishna R. Vaishnav A. Ade A. B.. (2022a). Biocontrol strategies: an eco-smart tool for integrated pest and diseases management. BMC Microbiol. 22, 1–5. 10.1186/s12866-022-02744-2 36581846 PMC9801620

[B16] Jaiswal D. K. Krishna R. Chouhan G. K. Araujo Pereira d. e. Ade A. P. Prakash A. B. J.. (2022b). Bio-fortification of minerals in crops: current scenario and future prospects for sustainable agriculture and human health. Plant Growth Regul. 98, 5–22. 10.1007/s10725-022-00847-4

[B17] Jaiswal D. K. Verma J. P. Krishna R. Gaurav A. K. Yadav J. (2019). Molecular characterization of monocrotophos and chlorpyrifos tolerant bacterial strain for enhancing seed germination of vegetable crops. Chemosphere 223, 636–650. 10.1016/j.chemosphere.2019.02.053 30798059

[B18] Jha M. Chourasia S. Sinha S. (2013). Microbial consortium for sustainable rice production. Agroecol. Sustain. Food Syst. 37, 340–362. 10.1080/10440046.2012.672376

[B19] Kaushal M. Devi S. Kumawat K. C. Kumar A. (2023). “Microbial consortium: a boon for a sustainable agriculture,” in Climate Change and Microbiome Dynamics: Carbon Cycle Feedbacks. (Cham: Springer International Publishing), pp. 15-31. 10.1007/978-3-031-21079-2_2

[B20] Khan A. Panthari D. Sharma R. S. Punetha A. Singh A. V. Upadhayay V. K.. (2023). “Biofertilizers: a microbial-assisted strategy to improve plant growth and soil health,” in Advanced Microbial Techniques in Agriculture, Environment, and Health Management. (Academic Press), pp. 97-118. 10.1016/B978-0-323-91643-1.00007-7

[B21] Kong Z. Hart M. Liu H. (2018). Paving the way from the lab to the field: using synthetic microbial consortia to produce high-quality crops. Front. Plant Sci. 9, 1467. 10.3389/fpls.2018.01467 30344529 PMC6182091

[B22] Krishna R. Jaiswal D. K. Ansari W. A. Singh S. Soumia P. S. Singh A. K.. (2022). Potential microbial consortium mitigates drought stress in tomato (*Solanum lycopersicum* L.) Plant by up-regulating stress-responsive genes and improving fruit yield and soil properties. J. Soil Sci. Plant Nutr. 3, 1–18. 10.1007/s42729-022-00929-2

[B23] Lebrun M. Michel C. Joulian C. Morabito D. Bourgerie S. (2021). Rehabilitation of mine soils by phytostabilization: does soil inoculation with microbial consortia stimulate Agrostis growth and metal (loid) immobilization?. Sci. Total Environ.791, 148400. 10.1016/j.scitotenv.2021.148400 34412406

[B24] Li X. Wu S. Fan H. Dong Y. Wang Y. Bai Z.. (2023). Phylogenetic distance affects the artificial microbial consortia's effectiveness and colonization during the bioremediation of polluted soil with Cr (VI) and atrazine. J. Hazard. Mater. 4, 131460. 10.1016/j.jhazmat.2023.131460 37141777

[B25] Liang Y. Ma A. Zhuang G. (2022). Construction of environmental synthetic microbial consortia: based on engineering and ecological principles. Front. Microbiol. 13, 829717. 10.3389/fmicb.2022.829717 35283862 PMC8905317

[B26] Liu X. Mei S. Salles J. F. (2023). Do inoculated microbial consortia perform better than single strains in living soil? A meta-analysis. BioRxiv 2023, 3. 10.1101/2023.03.17.533112

[B27] Mahreen N. Yasmin S. Asif M. Yahya M. Ejaz K. Yousaf S.. (2023). Mitigation of water scarcity with sustained growth of Rice by plant growth promoting bacteria. Front. Plant Sci. 14, 1081537. 10.3389/fpls.2023.1081537 36755700 PMC9900138

[B28] Mazzucotelli C. A. Durruty I. Kotlar C. E. Moreira M. D. R. Ponce A. G. Roura S. I.. (2014). Development of a microbial consortium for dairy wastewater treatment. Biotechnol. Bioprocess Eng. 19, 221–230. 10.1007/s12257-013-0517-8

[B29] Méndez-Bravo A. Herrera-Cornelio L. C. García-Toscano D. F. Kiel-Martínez A. L. Guevara-Avendaño E. Ramírez-Vázquez M.. (2023). Beneficial effects of selected rhizospheric and endophytic bacteria, inoculated individually or in combination, on non-native host plant development. Rhizosphere 26, 100693. 10.1016/j.rhisph.2023.10069327199897

[B30] Mukherjee A. Singh S. Gaurav A. K. Chouhan G. K. Jaiswal D. K. Araujo Pereira d. e. A. P.. (2022). Harnessing of phytomicrobiome for developing potential biostimulant consortium for enhancing the productivity of chickpea and soil health under sustainable agriculture. Sci. Total Environ. 836, 155550. 10.1016/j.scitotenv.2022.155550 35508232

[B31] Nabi M. (2023). Role of microorganisms in plant nutrition and soil health. In Sustainable Plant Nutrition (pp. 263-282). Academic Press. 10.1016/B978-0-443-18675-2.00016-X

[B32] Pankaj U. Pandey V. C. (Eds.). (2022). Microbial Based Land Restoration Handbook, Vol. 1: Plant-Microbial Interaction and Soil Remediation. CRC Press. 10.1201/9781003147091

[B33] Paravar A. Piri R. Balouchi H. Ma Y. (2023). Microbial seed coating: an attractive tool for sustainable agriculture. Biotechnol. Rep. 37, e00781. 10.1016/j.btre.2023.e00781 36655147 PMC9841043

[B34] Prajapati J. Yadav J. Jaiswal D. K. Prajapati B. Tiwari S. Yadav J.. (2022). Salt tolerant indigenous zn solubilizing bacteria isolated from forest organic soils promotes yield and root growth in oryza sativa under zinc deficient alluvial soil. Geomicrobiol. J. 39, 465–476. 10.1080/01490451.2022.2028941

[B35] Sarma B. K. Yadav S. K. Singh S. Singh H. B. (2015). Microbial consortium-mediated plant defense against phytopathogens: readdressing for enhancing efficacy. Soil Biol. Biochem. 87, 25–33. 10.1016/j.soilbio.2015.04.001

[B36] Shukla S. K. Sharma L. Jaiswal V. P. Pathak A. D. Tiwari R. Awasthi S. K.. (2020). Soil quality parameters vis-a-vis growth and yield attributes of sugarcane as influenced by integration of microbial consortium with NPK fertilizers. Sci. Rep.10, 19180. 10.1038/s41598-020-75829-5 33154431 PMC7645686

[B37] Silverstein M. R. Segrè D. Bhatnagar J. M. (2023). Environmental microbiome engineering for the mitigation of climate change. Glob. Change Biol. 29, 2050–2066. 10.1111/gcb.16609 36661406

[B38] Singh R. V. Kaur J. Bhagwat S. Arora Pandit M. Dogra Rawat C. (2023). Deploying microbes as drivers and indicators in ecological restoration. Restor. Ecol. 31, e13688. 10.1111/rec.13688

[B39] Singh J. S. Abhilash P. C. Gupta V. K. (2016). Agriculturally important microbes in sustainable food production. Trends Biotechnol. 34, 773–775. 10.1016/j.tibtech.2016.06.002

[B40] Verma J. P. Jaiswal D. K. Gaurav A. K. Mukherjee A. Krishna R. Araujo Pereira d. e. A. P.. (2023). Harnessing bacterial strain from rhizosphere to develop indigenous PGPR consortium for enhancing lobia (*Vigna unguiculata*) production. Heliyon 9, e13804. 10.1016/j.heliyon.2023.e13804 36895350 PMC9988462

[B41] Verma J. P. Jaiswal D. K. Sagar R. (2014b). Pesticide relevance and their microbial degradation: a-state-of-art. Rev. Environ. Sci. 13, 429–466. 10.1007/s11157-014-9341-7

[B42] Verma J. P. Yadav J. Tiwari K. N. Jaiswal D. K. (2014a). Evaluation of plant growth promoting activities of microbial strains and their effect on growth and yield of chickpea (*Cicer arietinum* L.) in India. Soil Biol. Biochem. 70, 33–37. 10.1016/j.soilbio.2013.12.001

[B43] Vishwakarma K. Kumar N. Shandilya C. Mohapatra S. Bhayana S. Varma A.. (2020). Revisiting plant–microbe interactions and microbial consortia application for enhancing sustainable agriculture: a review. Front. Microbiol. 11, 560406. 10.3389/fmicb.2020.560406 33408698 PMC7779480

[B44] Wu S. Li X. Fan H. Dong Y. Wang Y. Bai Z.. (2023). Engineering artificial microbial consortia based on division of labor promoted simultaneous removal of Cr (VI)-atrazine combined pollution. J. Hazard. Mater. 443, 130221. 10.1016/j.jhazmat.2022.130221 36367470

[B45] Yadav J. Verma J. P. Jaiswal D. K. Kumar A. (2014). Evaluation of PGPR and different concentration of phosphorus level on plant growth, yield and nutrient content of rice (*Oryza sativa*). Ecol. Eng. 62, 123–128. 10.1016/j.ecoleng.2013.10.013

[B46] Zhang C. Wu D. Ren H. (2020). Bioremediation of oil contaminated soil using agricultural wastes via microbial consortium. Sci. Rep. 10, 9188. 10.1038/s41598-020-66169-5 32513982 PMC7280287

